# Abattoirs

**Published:** 1881-05

**Authors:** 


ABATTOIRS.—A proper regard for the health of a city should be held of the highest importance by those intrusted with the management of its municipal affairs. Indeed, the ancient maxim, *Salus populi suprema lex*, should be held as important to us as it ever was to any, and should be implicitly obeyed. The march of improvement, or reform, is often slow, and not always sure in its results. But when a fact relating to the health of an entire municipality has been practically applied, and its usefulness clearly demonstrated and proven in a dozen instances, as in the establishment of abattoirs, it is certainly strange that the temporary loss of a few dollars and cents to a small class of its inhabitants should be permitted to influence or stand in the way of the abatement of a nuisance to the senses of all and a germinator of pestilence. No one who will pass by the slaughter-houses in our city of a warm day, or night either, can fail to perceive that they are the kind of nuisance we refer to. The erection of abattoirs beyond the city limits, and the abolishment of the slaughter-houses within the city, would no doubt operate to the temporary pecuniary disadvantage of the butchers, but they would suffer no serious injury from such a cause in the end, as has been proven by the experience of those cities in which abattoirs have been practically tested. The butchers in those cities have learned that their business can be carried on more profitably and satisfactorily under the abattoir system than it was when the private slaughter-house arrangement obtained.

We may have more to say on a future occasion on this subject, and must now be brief, that other matters may not be neglected.

